# Oxygenated Polyketides from *Plakinastrella mamillaris* as a New Chemotype of PXR Agonists

**DOI:** 10.3390/md11072314

**Published:** 2013-07-02

**Authors:** Carmen Festa, Claudio D’Amore, Barbara Renga, Gianluigi Lauro, Simona De Marino, Maria Valeria D’Auria, Giuseppe Bifulco, Angela Zampella, Stefano Fiorucci

**Affiliations:** 1 Department of Pharmacy, University of Naples “Federico II”, via D. Montesano 49, Naples 80131, Italy; E-Mails: carmen.festa@unina.it (C.F.); sidemari@unina.it (S.D.M.); madauria@unina.it (M.V.D.); 2 Department of Clinical and Experimental Medicine, Faculty of Medicine, University of Perugia, via Gerardo Dottori 1, S. Andrea Delle Fratte, Perugia 06132, Italy; E-Mails: claudiodamore1983@gmail.com (C.D.); barbara.renga@unipg.it (B.R.); fiorucci@unipg.it (S.F.); 3 Department of Pharmacy, University of Salerno, via Ponte don Melillo, Fisciano (SA) 84084, Italy; E-Mails: glauro@unisa.it (G.L.); bifulco@unisa.it (G.B.)

**Keywords:** marine sponge, *Plakinastrella mamillaris*, oxygenated polyketide, nuclear receptors, pregnane-X-receptor, anti-inflammatory activity

## Abstract

Further purification of the apolar extracts of the sponge *Plakinastrella mamillaris*, afforded a new oxygenated polyketide named gracilioether K, together with the previously isolated gracilioethers E–G and gracilioethers I and J. The structure of the new compound has been elucidated by extensive NMR (^1^H and ^13^C, COSY, HSQC, HMBC, and ROESY) and ESI-MS analysis. With the exception of gracilioether F, all compounds are endowed with potent pregnane-X-receptor (PXR) agonistic activity and therefore represent a new chemotype of potential anti-inflammatory leads. Docking calculations suggested theoretical binding modes of the identified compounds, compatible with an agonistic activity on hPXR, and clarified the molecular basis of their biological activities.

## 1. Introduction

Pregnane-X-receptor (PXR; NR1I2), a member of the nuclear receptor superfamily, primarily expressed in the liver and intestine [1], has a major role in the induction of genes involved in drug and xenobiotic transport and metabolism [2], thus preventing the accumulation of toxic chemicals in the body. In addition to its role as a xeno-sensor, PXR, through a complex, yet fully deciphered crosstalk with other nuclear receptors [3], such constitutive androstane receptor (CAR), farnesoid-X-receptor (FXR), liver-X-receptor (LXR), vitamin D receptor (VDR), peroxisome proliferator-activated receptors (PPARs), estrogen receptor (ER), glucocorticoid receptor (GR), COUP transcription factor I (COUP-TFI), and II, plays a relevant physiological role in the regulation of bilirubin clearance [4], bile acid detoxification [5,6,7], lipid, steroid [8], and glucose metabolism [9]. Moreover, recent studies demonstrated mutual inhibition between PXR and nuclear factor (NF)-κB signaling pathway, providing a potential molecular mechanism that links xenobiotic metabolism and inflammation [10,11]. These potential roles of PXR in disease pathogenesis suggest PXR as a novel target for drug discovery.

In recent years, several natural products from herbal medicine and marine sources were disclosed for their potentials to act as modulators of PXR [12,13,14]. In particular, studies from our laboratories disclosed some sterols [15,16,17,18] from the marine sponge *Theonella swinhoei* as a new class of PXR agonists that exerted potent immunomodulatory activity. Extensive *in vivo* and *in vitro* studies on solomonsterol A confirmed the therapeutic potential of PXR agonists in the treatment of inflammatory bowel disease (IBD) [19]. Further characterization of natural PXR agonists, and their biological activities, may hold the promise of developing novel PXR modulators for the treatment of metabolic diseases.

A recent report [20] described ginkgolides, terpene trilactones present in *Ginkgo biloba* extract, as a new chemotype for ligand activation of hPXR.

**Figure 1:**
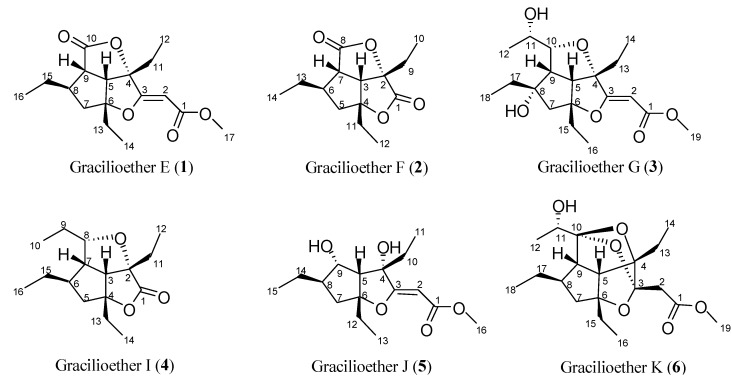
Gracilioethers from the sponge *Plakinastrella mamillaris*.

In connection with our interest toward the discovery of new ligands for nuclear receptors, we were intrigued by the structural resemblance between the polycyclic “cage-like” backbone of ginkgolides with the oxygenated polycyclic structure of gracilioethers E, F, G, I, and J (**1**–**5**, Figure 1), polyketides recently isolated by our group [21]. 

Therefore docking studies were performed to assess the accommodation of gracilioethers in the ligand binding domain (LBD) of hPXR, and to highlight the pharmacophoric parts able to establish weak interactions with the key residues of the investigated target. 

In order to re-obtain the gracilioethers for pharmacological evaluations on the pure components of the apolar extracts of the sponge *Plakinastrella mamillaris*, a new oxygenated polyketide, named gracilioether K (**6**), was also isolated.

In the present study we reported the results of the docking analysis and the pharmacological evaluation indicating that, with the exception of gracilioether F (**2**), all compounds are endowed with potent PXR agonistic activity. 

The structural characterization of the new polyketide derivative, gracilioether K, is also reported.

## 2. Results and Discussion

The starting hypothesis of considering gracilioethers **1**–**5**, as characterized by structural requirements compatible with an agonistic activity on hPXR, was investigated by means of molecular docking experiments. As previously described [22,23,24], a putative PXR ligand should be able to interact with polar residues in the LBD. In this context, Ekins *et al*. proposed several agonist pharmacophoric models, characterized by the presence of a hydrogen bond acceptor and/or donor, and hydrophobes [25]. In particular Ser247 and other polar residues (namely Cys284, Gln285, His407) have been reported as key amino acids involved in hydrogen bond/polar interactions with the ligand counterpart.

**Figure 2:**
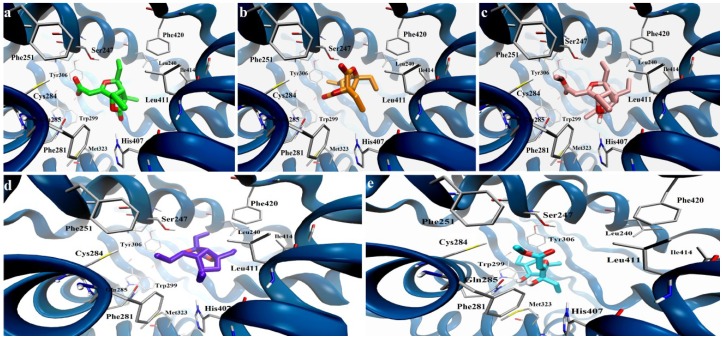
Docking model of gracilioethers **1**–**5** in the ligand binding domain (LBD) of hPXR (secondary structure represented in blue): (**a**) **1** (pose n. 4, colored by atom types: C green, O red); (**b**) **2** (pose n. 27, colored by atom types: C orange, O red); (**c**) **3** (pose n. 2, colored by atom types: C pink, O red, H light grey); (**d**) **4** (pose n. 11, colored by atom types: C violet, O red); (**e**) **5** (pose n. 1, colored by atom types: C cyan, O red, H light grey).

From a structural point of view, gracilioethers **1**–**5** are characterized by a well defined region with a high density of oxygens, potentially able to establish hydrogen bonds and polar interactions, and by a spatially distinct hydrophobic part. Starting from these assumptions, we have envisaged a binding mode, in which a part of these molecules is involved in polar interactions and/or hydrogen bonds with the key residues in the hPXR LBD, while the remaining part stabilizes the complex by van der Waals interactions (Figure 2). For each investigated compound, the docking analysis of the different binding poses was then conducted using a distance criterion, filtering and considering the number of poses interacting with key residues, Ser247, Cys284, Gln285, and His407.

Firstly, this analysis highlighted the ability of the investigated compounds to interact with the Ser247 [24] via polar interactions or hydrogen bonds in a high number of docking poses. In particular, for gracilioethers **1**, **3**, and **5** we found these fundamental interactions in more than 55% of the 50 docking poses saved for each compound. The significant number of polar groups in a defined part of their structures determines the stabilization of these putative complexes in the hPXR LBD through further hydrogen bonds or polar interactions (Gln285, Cys284, and His407), while the hydrophobic components are involved in van der Waals contacts (namely Leu240, Phe251, Phe281, Ile414). 

If compared to compounds **1**, **3**, and **5**, gracilioethers **2** and **4** are characterized by the absence of the unsaturated side chain at C-3, determining a minor number of interactions with the above reported key residues. Interestingly, a smaller percentage of poses interacting with Ser247 were obtained (30% for compound **2** and 36% for compound **4**). A sensible minor number of matching poses were found for the interactions with Cys284 and His407, respecting the relative distributions between the different compounds. 

The main difference between these two compounds is the presence of an additional ethyl group at C-8 in compound **4** replacing the γ-lactone moiety in compound **2**. Analyzing the poses of **4** interacting with Ser247, we noticed that the presence of this aliphatic part in compound **4** determines the gain of favorable van der Waals interactions (Leu240, Phe251, Phe281, Leu411, Ile414) [24]. 

On the other hand, the analysis of the complexes of **2** interacting with Ser247 of hPXR revealed that the carbonyl part of the γ-lactone moiety is scarcely involved in hydrogen bonds or favorable polar interactions with the other key residues previously described. In addition, with respect to **4**, the hPXR complex with gracilioether F (**2**) is characterized by poor hydrophobic contacts. An opposite situation was observed for gracilioether E (**1**), also featuring the γ-lactone moiety, where this chemical group contributes to the above polar pharmacophoric interactions in the most energetically favored poses. 

These differences are depicted in Figure 2, where a representation of the binding modes of the different gracilioethers is reported. In these images, the poses in which the common chemical parts among these compounds occupy the same region in the hPXR LBD in a similar way are displayed; in such poses, the requirements of a putative agonistic activity, polar interactions with Ser247 for all compounds and with Gln285 for **1**–**3** and **5**, are maintained. Moreover, the reported poses highlight that the punctual differences in the chemical structures of gracilioethers could determine the loss or the gain of additional hydrophobic (e.g., Phe251, Phe281, Ile414 found in compound **4**, absent in **2**) or polar interactions (His407), also influencing the stabilization of the corresponding hPXR LBD complexes and, consequently, the biological activity.

### 2.1. Isolation of Gracilioethers and Structural Characterization of Gracilioether K *(**6**)*

Prompted by good docking results, a re-isolation procedure was performed on the CHCl_3_ extract of the sponge *Plakinastrella mamillaris*. MPLC followed by HPLC purification afforded **1**–**5** in reasonable amounts for further pharmacological tests and the new oxygenated polyketide named gracilioether K (**6**).

Gracilioether K (**6**) was isolated as a white amorphous solid. The molecular formula of C_19_H_30_O_6_ was established by HR-ESIMS based on the pseudomolecular ion [M + Na]^+^ at *m*/*z* 377.1954, indicating an isomeric relationship with gracilioethers A [26] and G (**3**) [21] and five degrees of unsaturation.

Analysis of the ^1^H NMR and HSQC spectra revealed the presence of three ethyls, one methoxyl, one methyl, four methines (including one oxymethine), and two methylenes (Table 1). The ^13^C NMR spectrum further showed the presence of one acyl carbon at δ_C_ 171.1, two oxygenated quaternary carbons at δ_C_ 93.8 and 96.7, and two ketalic carbons at δ_C_ 110.49 and 110.51 (Table 1). 

**Table 1:** ^1^H and ^13^C NMR data (700 and 175 MHz, CD_3_OD) of gracilioether K (**6**).

Position	δ_H _^a^	δ_C_	HMBC	ROESY ^b^
1	-	171.1		
2	2.63 d (14.2) 2.71 d (14.2)	40.2	C1, C3	H_2_-13, H_3_-16
3	-	110.49		
4	-	96.7		
5	2.80 d (10.5)	54.6	C3, C6, C9, C15	H-7b, H_2_-13, H_3_-14, H_2_-15, H_3_-16
6	-	93.8		
7	1.51 m 2.28 dd (7.6, 13.8)	47.9	C5, C6, C8, C9	H-5, H-9, H_3_-16
8	2.06 ovl	39.4		
9	2.74 dd (3.7, 10.5)	59.0		H-7b, H_3_-12, H_2_-17, H_3_-18
10	-	110.51		
11	3.88 q (6.5)	66.0	C10	
12	1.22 d (6.5)	17.7	C10, C11	H-9, H_3_-18
13	1.74 quint (7.5) 2.07 ovl	21.9	C4, C14 C3, C4, C5	H_2_-2, H-5 H_2_-2
14	1.07 t (7.4)	9.1	C4	H-5
15	1.55 m 1.65 quint (7.3)	32.6	C5, C7	H-5 H-5
16	0.93 t (7.5)	9.8	C6	H_2_-2, H-5, H-7b
17	1.32 m 1.53 m	30.5	C7, C8, C9, C18	H-9 H-9
18	0.91 t (7.4)	12.9	C8	H-9, H_3_-12
19	3.62 s	52.2	C1	

^a^ Coupling constants are in parentheses and given in hertz. ^1^H and ^13^C assignments aided by COSY, HSQC, and HMBC experiments; ^b^ ROESY experiment with mixing time of 200 ms; ovl: overlapped with other signals.

A detailed analysis of 2D NMR data indicated a carbon framework for gracilioether K (**6**) very similar to that of gracilioether A [26]. The most significant difference is the loss, in gracilioether K, of two sp^2^ carbons (δ_H/C_ 4.86/86.8 and δ_C_ 174.6), assigned respectively at C-2 and C-3 in gracilioether A [26].

**Figure 3:**
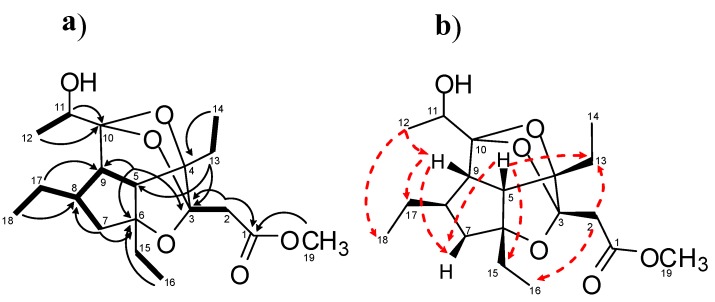
(**a**) COSY connectivities (bold bonds), and key HMBC (black arrows) correlations; (**b**) Key ROE (red dashed arrows) correlations for gracilioether K (**6**).

Analysis of COSY data allowed to the identify five spin systems: Two ethyl groups linked to quaternary carbons, a CH(5)CH(9)CH(8)(CH_2_CH_3_)CH_2_(7), a CH_3_(12)CH(11)OH, and one isolated CH_2_(2) (δ_H_ 2.63 and 2.71) spin systems (Figure 3a and Table 1). The HMBC correlations H_3_-14/C-4 and H_3_-16/C-6 connected the two ethyl units to C-4 and C-6 quaternary carbons, respectively. The hydroxyethyl group, CH_3_(12)CH(11)OH, was connected to the ketalic quaternary carbon C-10 (δ_C_ 110.51) on the basis of HMBC correlations H_3_-12/C-10 and H-11/C-10. The isolated AB spin system (δ_H_ 2.63 and 2.71) was connected to the acyl group at δ_C_ 171.1 and to the ketal carbon at δ_C_ 110.49 on the basis of HMBC correlations H-2/C-1 and H-2/C-3. A methyl ester group was easily inferred by the diagnostic long-range correlation between the methoxy protons at δ_H_ 3.62 and the acyl carbonyl at δ_C_ 171.1. Several diagnostic HMBC correlations (Figure 3a and Table 1) allowed us to secure the presence of the hexahydro-cyclopentanfurane system common to all gracilioethers, in which an ether bridge connecting C-3 and C-6 was placed by analogy with other gracilioethers and chemical shift considerations. Although no diagnostic heteronuclear long-range correlations were found to prove the C-9/C-10 linkage, the attachment of C-10/C-12 subunit to C-9 was supposed by analogy with gracilioether A, and confirmed by the strong dipolar couplings between H_3_-12/H-9 and H_3_-18 (Figure 3b).

Finally, two ether bridges between C-3 and C-10 and between C-4 and C-10 were assigned to satisfy the degrees of unsaturation and the molecular formula and to fulfil the functionalization degree of C-3, C-4, and C-10, as determined by ^13^C NMR chemical shifts value analysis.

The relative configuration of the tetracyclic system in **6** was established from ROESY correlations (Table 1), as shown in Figure 3b. The correlations between H-5/H-7b, H-13, H-14, H-15, H-16, and H-9/H-7b, H-17, H-18 suggested that the three ethyl groups, H-5 and H-9, were on the same face of the rings in a similar situation of all members of this family of marine oxygenated polyketides [21,26]. 

The analysis of the ROESY spectrum gave also information on the stereochemistry at C-3. In particular ROESY correlations H-2/H_2_-13 and H_3_-16 indicated that the carbomethoxy methyl substituent at C-3 and the ethyl group at C-4 were *cis*-oriented.

**Figure 4:**
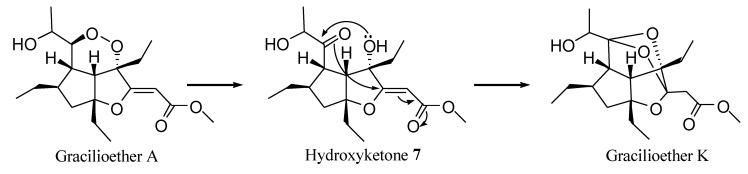
Biosynthetic pathway to gracilioether K, involving endoperoxy rearrangement, hemiacetal formation and Michael-type attack on the α,β-unsaturated ester group.

The stereochemical homology observed for all members of the gracilioethers family previously isolated from the sponge *Plakinastrella mamillaris* [21,26] suggested a common biogenetic origin. By analogy with well known biosynthetic pathway to PGE_2_, endoperoxy rearrangement in gracilioether A could afford hydroxyketone **7** that, through hemiacetal ring closure followed by 1,4 nucleophilic addition of the C-10 oxygen group to the unsaturated ester moiety, generated the complex polycyclic system of gracilioether K (Figure 4). 

Therefore, from this biogenetic relationship, gracilioether K is likely to retain the same absolute configuration of all stereogenic centers of the hexahydro-cyclopentanfurane system as in gracilioether A [26].

**Figure 5:**
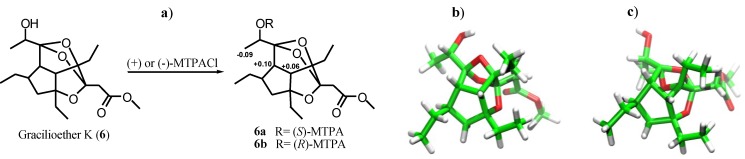
(**a**) ∆δ*_S_*_-*R*_ values (ppm) of MTPA esters **6a** and **6b**; 3D model for (**b**) 10*R* configuration and (**c**) 10*S* configuration.

The absolute stereochemistry of the secondary hydroxyl group at C-11 was determined by application of the modified Mosher’s method [27]. Treatment of **6** with *R*-(**−**)- or *S*-(+)-α-methoxy-α-(trifluoromethyl)phenylacetyl chloride (MTPACl) yielded *S*-(**−**)- and *R*-(+)-MTPA esters **6a** and **6b**, respectively. The ∆δ value distribution pattern clearly indicated 11*S* configuration (Figure 5a), the same found in gracilioether G (**3**) [21].

Finally, once fixed the configuration of all the other stereogenic centers, the analysis of the two possible diasteroisomeric structures presenting the 10*R* (Figure 5b) or 10*S* (Figure 5c) configuration revealed the presence of severe unfavorable distortions of the sp^3^ atoms involved in the interested rings for the *S* configuration at C-10 thus suggesting the *R* configuration as depicted in Figure 1. 

### 2.2. Pharmacological Evaluation

**Figure 6:**
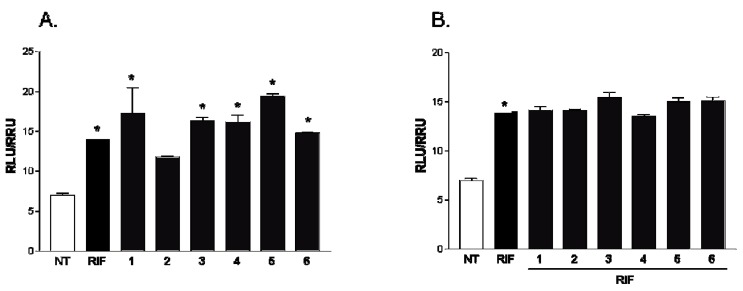
Luciferase reporter assay performed in HepG2 transiently transfected with pSG5-PXR, pSG5-RXR, pCMV-bgal, p(cyp3a4)TKLUC vectors and stimulated 18 h with (**A**) Rifaximin (RIF), 10 μM, and of gracilioethers **1**–**6**, 10 μM; and (**B**) Rifaximin (RIF), 10 μM, alone or in combination with gracilioethers **1**–**6**, 50 μM. * *p* < 0.05 *vs.* not treated (NT).

**Figure 7:**
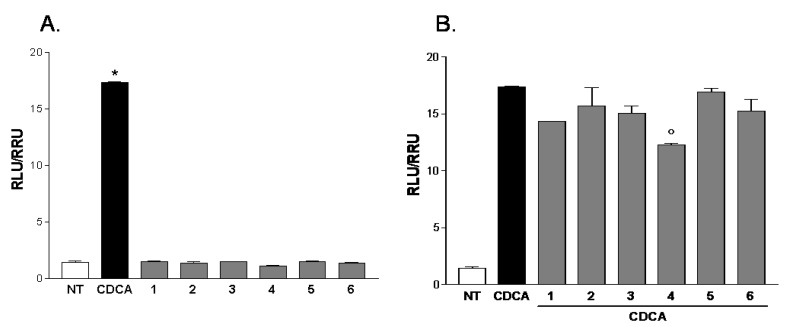
Luciferase reporter assay performed in HepG2 transiently transfected with pSG5-FXR, pSG5-RXR, pCMV-bgal, p(hsp27)TKLUC vectors and stimulated 18 h with (**A**) CDCA, 10 μM, and gracilioethers **1**–**6**, 10 μM; and (**B**) CDCA, 10 μM, alone or in combination with gracilioethers **1**–**6**. * *p* < 0.05 *vs.* not treated (NT), ° *p* < 0.05 *vs.* CDCA.

All compounds of this small series were challenged in a reporter gene assay using HepG2 cells, a human hepatoma cell line. As shown in Figure 6, all compounds, with the exception of gracilioether F (**2**), were PXR agonists without effect when administered in combination with rifaximin. However, when HepG2 cells transfected with FXR vectors were treated with gracilioethers **1**–**6**, alone or in the presence of chenodeoxycholic acid (CDCA) 10 μM, no significant agonistic or antagonistic activities were observed, with the exception for the compound **4**, which revealed a slight antagonistic activity on FXR (Figure 7).

**Figure 8:**
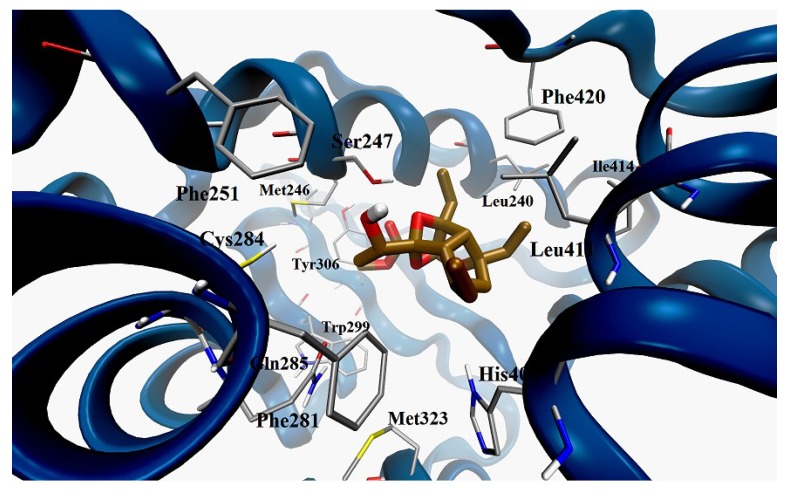
Docking model of gracilioether K (**6**) (pose n. 2, colored by atom types: C ochre, O red, H light grey); in the LBD of hPXR (secondary structure represented in blue).

Docking calculations on graciolioether K (**6**) confirmed the observations highlighted for active gracilioethers **1**, **3**, **4** and **5**. As shown in Figure 8, graciolioether K (**6**) establishes polar contacts with Ser247 and His407, and van der Waals interactions with Met243, Leu411, Ile414, and Phe420.

## 3. Experimental Section

### 3.1. General Experimental Procedures

Specific rotations were measured on a Perkin-Elmer 243 B polarimeter. High-resolution ESI-MS measurements were performed with a Micromass QTOF Micromass spectrometer. ESI-MS experiments were performed on an Applied Biosystem API 2000 triple-quadrupole mass spectrometer. NMR spectra were obtained on a Varian Inova 700 MHz spectrometer (^1^H at 700 MHz, ^13^C at 175 MHz, respectively) equipped with Sun hardware, δ (ppm), *J* in Hz, spectra referred to CD_3_OD (δ_H_ 3.31, δ_C_ 49.0) as internal standards. HPLC was performed using a Waters Model 510 pump equipped with Waters Rheodyne injector and a differential refractometer, model 401. Through-space ^1^H connectivities were evidenced using a ROESY experiment with a mixing time of 200 ms. Silica gel (200–400 mesh) from Macherey-Nagel Company (Düren, Germany) was used for flash chromatography. The purities of compounds were determined to be greater than 95% by HPLC. 

### 3.2. Sponge Material and Separation of Gracilioethers

*Plakinastrella mamillaris* Kirkpatrick, 1900 (order Homosclerophorida, family Plakinidae) was collected at the Fiji Islands, in May 2007. The sample was frozen immediately after collection and lyophilized to yield 171 g of dry mass. The sponge was identified by John Hooper, Queensland Museum, Brisbane, Australia, where a voucher specimen is deposited under the accessing number G322695. 

The lyophilized material (171 g) was extracted with methanol (3 × 1.5 L) at room temperature and the crude methanolic extract (40 g) was subjected to a modified Kupchan’s partitioning procedure as follows. The methanol extract was dissolved in a mixture of MeOH/H_2_O containing 10% H_2_O and partitioned against *n*-hexane to give 17.3 g of the crude extract. The water content (% v/v) of the MeOH extract was adjusted to 30% and partitioned against CHCl_3_ to give 16.6 g of the crude extract.

The aqueous phase was concentrated to remove MeOH and then extracted with *n*-BuOH (2.4 g of crude extract). The CHCl_3_ extract (4.0 g) was chromatographed by silica gel MPLC using a solvent gradient system from CH_2_Cl_2_ to CH_2_Cl_2_:MeOH 1:1. Fractions eluted with CH_2_Cl_2_:MeOH 99:1 (370 mg) were further purified by HPLC on a Nucleodur 100-5 C_18_ (5 μm; 10 mm i.d. × 250 mm) with 65% MeOH:H_2_O as eluent (flow rate 3.5 mL/min) to give 5.3 mg of gracilioether K (**6**) (*t*_R_ = 44.1 min); 70% MeOH:H_2_O as eluent (flow rate 3.5 mL/min) to give 3.3 mg of gracilioether F (**2**) (*t*_R_ = 9.6 min), 6.1 mg of gracilioether E (**1**) (*t*_R_ = 11.1 min), 11.0 mg of gracilioether A (*t*_R_ = 26 min), 0.9 mg of gracilioether I (**4**) (*t*_R_ = 27.9 min). Fractions eluted with CH_2_Cl_2_/MeOH 98:2 (326 mg) were further purified by HPLC on a Nucleodur 100-5 C_18_ (5 μm; 10 mm i.d. × 250 mm) with 60% MeOH/H_2_O as eluent (flow rate 3.5 mL/min) to give 4.3 mg of gracilioether J (**5**) (*t*_R_ = 12.6 min) and 8.5 mg of gracilioether G (**3**) (*t*_R_ = 21.9 min).

### 3.3. Characteristic Data for Gracilioether K *(**6**)*

Gracilioether K (**6**): White amorphous solid; [α]_D_^25^ −32.1 (*c* 0.06, MeOH); ^1^H and ^13^C NMR data in CD_3_OD given in Table 1; ESI-MS: *m*/*z* 377.2 [M + Na]^+^. HRMS (ESI): calcd. for C_19_H_30_NaO_6_: 377.1940; found 377.1954 [M + Na]^+^.

### 3.4. General Procedure for the Preparation of MTPA Esters (**6a**) and (**6b**)

Samples of 0.5 mg were dissolved in freshly distilled CH_2_Cl_2_ and treated with triethylamine (10 μL), (*R*)- or (*S*)-α-methoxy-α-(trifluoromethyl)phenylacetyl chloride (MTPA-Cl) (5 μL) and a catalytic amount of 4-(dimethylamino)pyridine to obtain respectively (*S*)- or (*R*)-MTPA esters. The mixture was left to stand at room temperature for 1 h, with the resulting mixture purified by HPLC on a Luna 5 μ Silica (2) column (5 μm; 4.6 mm i.d. × 250 mm) with 70% hexane:EtOAc as eluent (flow rate 1.0 mL/min). 

[(*S*)-MTPA ester **6a**]: Selected ^1^H NMR (400 MHz, CD_3_OD) δ (ppm): 1.29 (d, *J* = 6.3 Hz, H_3_-12), 1.51 (m, H-7a), 2.23 (dd, *J* = 7.9, 13.8 Hz, H-7b), 2.49 (dd, *J* = 3.6, 10.4 Hz, H-9), 2.81 (d, *J* = 10.4 Hz, H-5), 5.34 (q, *J* = 6.3 Hz, H-11). 

[(*R*)-MTPA ester **6b**]: Selected ^1^H NMR (400 MHz, CD_3_OD) δ (ppm): 1.38 (d, *J* = 6.5 Hz, H_3_-12), 1.48 (m, H-7a), 2.12 (dd, *J* = 8.1, 13.6 Hz, H-7b), 2.39 (dd, *J* = 3.6, 10.3 Hz, H-9), 2.75 (d, *J* = 10.3 Hz, H-5), 5.35 (q, *J* = 6.5 Hz, H-11).

### 3.5. Computation Details

The chemical structures of the compounds were built and processed with Macromodel 8.5 (Schrödinger, LLC, New York, NY, USA, 2003). Conformational search and Molecular Dynamics calculations were performed on a 4× AMD Opteron SixCore 2.4 GHz using Macromodel 8.5. Conformational search simulations were performed using the OPLS_2005 force field. The Monte Carlo multiple minimum (MCMM) method (10^5^ steps) was used to allow a full exploration of the conformational space. Molecular Dynamics simulations were performed at 450 K and with a simulation time of 10 ns, after an equilibration phase of 1 ns. A constant dielectric term, mimicking the presence of the solvent, was used to reduce artifacts. Finally, the optimization (Conjugate Gradient, 0.0005 Å convergence threshold) of the structures was applied to identify the three-dimensional starting models for the subsequent steps of docking calculations. 

#### Docking Calculation

Docking calculations were performed using the Autodock-Vina software [28]. In the configuration files of the crystallographic structure of hPXR (PDB code: 1M13) we specified the coordinate values for the targets, focusing the grid on the site of presumable pharmacological interest. In particular a grid box size of 30 × 36 × 32 and centered at 14.282 (*x*), 74.983 (*y*), and 0.974 (*z*) was used, with spacing of 1.0 Å between the grid points. The exhaustiveness value was set to 64, saving 50 conformations as maximum number of binding modes, and choosing 3.0 kcal/mol as maximum energy difference between the best and worst binding pose calculated. For all the investigated compounds, all open-chain bonds were treated as active torsional bonds. Docking results were analyzed with Autodock Tools 1.4.5. Illustrations of the 3D models were generated using VMD software [29].

### 3.6. *In Vitro* Transactivation

HepG2 cells were cultured at 37 °C in Minimum Essential Medium with Earl’s salts containing 10% fetal bovine serum (FBS), 1% l-glutamine and 1% penicillin/streptomycin. HepG2 cells were plated in a 24-wells plate at 1 × 10^5^ cells/well. The transfection experiments were performed using Fugene HD (Promega, Milan, Italy) according to manufacturer specifications. For FXR mediated transactivation, cells were transfected with 75 ng pCMVSPORT-FXR, 75 ng pSG5-RXR, 100 ng pGL4.70-Renilla and with 250 ng of the reporter vector p(hsp27)-TK-LUC containing the FXR response element IR1 cloned from the promoter of heat shock protein 27 (hsp27). For PXR mediated transactivation, cells were transfected with 75 ng pSG5-hPXRT1, 75 ng pSG5-RXR, 100 ng pGL4.70-Renilla, and with 250 ng of the reporter vector pGL3(henance)PXRE. At 24 h post-transfection, cells were treated for 2 h with compounds **1**–**6** 10 μM (agonism) or 50 μM (antagonism) and then stimulated with CDCA 10 μM for 18 h. After treatments, cells were lysed in 100 μL diluted reporter lysis buffer (Promega) and 10 μL cellular lysate was assayed for Luciferase and Renilla activity using the Luciferase or Renilla Assay System (Promega). Luminescence was measured using GloMax™ 20/20 Luminometer (Promega). Luciferase activities were normalized for transfection efficiencies by dividing the Luciferase relative light units by Renilla relative lights units expressed from cells co-transfected with pGL4.70-Renilla. 

## 4. Conclusions

The demonstration that gracilioethers, with the exception of gracilioether F, are PXR agonists, devoid of a significant activity on the bile acid sensor FXR, might have pharmacological and therapeutic relevance. Indeed, PXR agonists are being increasingly investigated for their ability to reduce inflammation in gastrointestinal tract and for their potential application in the treatment of a variety of human disorders including altered bone homeostasis, liver steatosis, and liver and lung fibrosis. PXR has, additionally, a multi-factorial impact on cancer, either by directly affecting cell proliferation and apoptosis, or by inducing chemotherapy resistance in colon, breast, prostate, and endometrial cancer, and in osteosarcoma, suggesting that identification of novel PXR ligands, agonists and antagonists, might have relevance for the treatment of these disorders. This study reaffirms sponges of *Plakinastrella* genus as an invaluable source of nuclear receptors ligands [30] endowed with unusual chemical scaffolds and useful for discovery of new pharmacological leads in several human diseases. 

## References

[1] Lehmann J.M., McKee D.D., Watson M.A., Willson T.M., Moore J.T., Kliewer S.A. (1998). The human orphan nuclear receptor PXR is activated by compounds that regulate CYP3A4 gene expression and cause drug interactions. J. Clin. Invest..

[2] Kliewer S.A., Goodwin B., Willson T.M. (2002). The nuclear pregnane X receptor: A key regulator of xenobiotic metabolism. Endocr. Rev..

[3] Reschly E.J., Krasowski M.D. (2006). Evolution and function of the NR1I nuclear hormone receptor subfamily (VDR, PXR, and CAR) with respect to metabolism of xenobiotics and endogenous compounds. Curr. Drug Metab..

[4] Saini S.P., Mu Y., Gong H., Toma D., Uppal H., Ren S., Li S., Poloyac S.M., Xie W. (2005). Dual role of orphan nuclear receptor pregnane X receptor in bilirubin detoxification in mice. Hepatology.

[5] Xie W., Radominska-Pandya A., Shi Y., Simon C.M., Nelson M.C., Ong E.S., Waxman D.J., Evans R.M. (2001). An essential role for nuclear receptors SXR/PXR in detoxification of cholestatic bile acids. Proc. Natl. Acad. Sci. USA.

[6] Staudinger J.L., Goodwin B., Jones S.A., Hawkins-Brown D., MacKenzie K.I., LaTour A., Liu Y., Klaassen C.D., Brown K.K., Reinhard J. (2001). The nuclear receptor PXR is a lithocholic acid sensor that protects against liver toxicity. Proc. Natl. Acad. Sci. USA.

[7] Fiorucci S., Cipriani S., Baldelli F., Mencarelli A. (2010). Bile acid-activated receptors in the treatment of dyslipidemia and related disorders. Prog. Lipid Res..

[8] Sonoda J., Chong L.W., Downes M., Barish G.D., Coulter S., Liddle C., Lee C.H., Evans R.M. (2005). Pregnane X receptor prevents hepatorenal toxicity from cholesterol metabolites. Proc. Natl. Acad. Sci. USA.

[9] Ihunnah C.A., Jiang M., Xie W. (2011). Nuclear receptor PXR, transcriptional circuits and metabolic relevance. Biochim. Biophys. Acta.

[10] Xie W., Tian Y. (2006). Xenobiotic receptor meets NF-kappaB, a collision in the small bowel. Cell Metab..

[11] Zhou C., Tabb M.M., Nelson E.L., Grun F., Verma S., Sadatrafiei A., Lin M., Mallick S., Forman B.M., Thummel K.E. (2006). Mutual repression between steroid and xenobiotic receptor and NF-kappaB signaling pathways links xenobiotic metabolism and inflammation. J. Clin. Invest..

[12] D’Auria M.V., Sepe V., Zampella A. (2012). Natural ligands for nuclear receptors: Biology and potential therapeutic applications. Curr. Top. Med. Chem..

[13] Gao J., Xie W. (2012). Targeting xenobiotic receptors PXR and CAR for metabolic diseases. Trends Pharmacol. Sci..

[14] Fiorucci S., Distrutti E., Bifulco G., D’Auria M.V., Zampella A. (2012). Marine sponge steroids as nuclear receptor ligands. Trends Pharmacol. Sci..

[15] De Marino S., Ummarino R., D’Auria M.V., Chini M.G., Bifulco G., Renga B., D’Amore C., Fiorucci S., Debitus C., Zampella A. (2011). Theonellasterols and conicasterols from *Theonella swinhoei*. Novel marine natural ligands for human nuclear receptors. J. Med. Chem..

[16] De Marino S., Ummarino R., D’Auria M.V., Chini M.G., Bifulco G., D’Amore C., Renga B., Mencarelli A., Petek S., Fiorucci S. (2012). 4-Methylenesterols from *Theonella swinhoei* sponge are natural pregnane-X-receptor agonists and farnesoid-X-receptor antagonists that modulate innate immunity. Steroids.

[17] Festa C., de Marino S., D’Auria M.V., Bifulco G., Renga B., Fiorucci S., Petek S.,  Zampella A. (2011). Solomonsterols A and B from *Theonella swinhoei*. The first example of C-24 and C-23 sulfated sterols from a marine source endowed with a PXR agonistic activity. J. Med. Chem..

[18] De Marino S., Sepe V., D’Auria M.V., Bifulco G., Renga B., Petek S., Fiorucci S.,  Zampella A. (2011). Towards new ligands of nuclear receptors. Discovery of malaitasterol A, an unique *bis*-secosterol from marine sponge *Theonella swinhoei*. Org. Biomol. Chem..

[19] Sepe V., Ummarino R., D’Auria M.V., Mencarelli A., D’Amore C., Renga B., Zampella A., Fiorucci S. (2011). Total synthesis and pharmacological characterization of solomonsterol A, a potent marine pregnane-X-receptor agonist endowed with anti-inflammatory activity. J. Med. Chem..

[20] Lau A.J., Yang G., Yap C.W., Chang T.K.H. (2012). Selective agonism of human pregnane X receptor by individual ginkgolides. Drug Metab. Dispos..

[21] Festa C., de Marino S., D’Auria M.V., Deharo E., Gonzalez G., Deyssard C., Petek S., Bifulco G., Zampella A. (2012). Gracilioethers E–J, new oxygenated polyketides from the marine sponge *Plakinastrella mamillaris*. Tetrahedron.

[22] Ekins S., Kortagere S., Iyer M., Reschly E.J., Lill M.A., Redinbo M.R., Krasowski M.D. (2009). Challenges predicting ligand-receptor interactions of promiscuous proteins: The nuclear receptor PXR. PLoS Comput. Biol..

[23] Watkins R.E., Maglich J.M., Moore L.B., Wisely G.B., Noble S.M., Davis-Searles P.R., Lambert M.H., Kliewer S.A., Redinbo M.R. (2003). 2.1 Å crystal structure of human PXR in complex with the St. John’s wort compound hyperforin. Biochemistry.

[24] Xiao L., Nickbarg E., Wang W., Thomas A., Ziebell M., Prosise W.W., Lesburg C.A., Taremi S.S., Gerlach V.L., Le H.V. (2011). Evaluation of *in vitro* PXR-based assays and in silico modeling approaches for understanding the binding of a structurally diverse set of drugs to PXR. Biochem. Pharmacol..

[25] Ekins S., Chang C., Mani S., Krasowski M.D., Reschly E.J., Iyer M., Kholodovych V., Ai N., Welsh W.J., Sinz M. (2007). Human pregnane X receptor antagonists and agonists define molecular requirements for different binding sites. Mol. Pharmacol..

[26] Ueoka R., Nakao Y., Kawatsu S., Yaegashi J., Matsumoto Y., Matsunaga S., Furihata K., van Soest R.W.M., Fusetani N. (2009). Gracilioethers A–C, antimalarial metabolites from the marine sponge *Agelas gracilis*. J. Org. Chem..

[27] Ohtani I., Kusumi T., Kashman Y., Kakisawa H. (1991). High-field FT NMR application of Mosher’s method. The absolute configurations of marine terpenoids. J. Am. Chem. Soc..

[28] Trott O., Olson A.J. (2010). AutoDock Vina: Improving the speed and accuracy of docking with a new scoring function, efficient optimization and multithreading. J. Comput. Chem..

[29] Humphrey W., Dalke A., Schulten K. (1996). VMD—Visual molecular dynamics. J. Mol. Graph..

[30] Festa C., Lauro G., de Marino S., D’Auria M.V., Monti M.C., Casapullo A.,  D’Amore C., Renga B., Mencarelli A., Petek S. (2012). Plakilactones from the marine sponge *Plakinastrella mamillaris*. Discovery of a new class of marine ligands of peroxisome proliferator-activated receptor γ. J. Med. Chem..

